# LncRNA MAGI2-AS3 is involved in cervical squamous cell carcinoma development through CDK6 up-regulation

**DOI:** 10.1186/s13027-019-0238-5

**Published:** 2019-11-20

**Authors:** Qifang Liu, Shuang Liu, Xiaoying Wang, Jin Zhang, Kuiran Liu

**Affiliations:** Department of obstetrics and gynecology, Shengjing hospital affiliated to China medical university, No. 36 of Sanhao Street, Heping District, Shenyang, 110004 Liaoning China

**Keywords:** Cervical squamous cell carcinoma, lncRNA MAGI2-AS3, CDK6, Prognosis

## Abstract

**Background:**

It has been reported that lncRNA MAGI2-AS3 can promote many types of cancer, such as breast cancer and bladder cancer, by regulating cell behaviors, such a proliferation, invasion, and migration. However, its role in cervical squamous cell carcinoma (CSCC) is unclear. This study aims to investigate the role of MAGI2-AS3 in CSCC.

**Methods:**

Sixty-four CSCC patients (36 to 68 years, 46.3 ± 5.1 years) out of 136 CSCC patients admitted by Shengjing hospital affiliated to China Medical University from June 2010 to October 2013 were included in the present study. Among the 64 enrolled patients, 20 were HPV-16 positive cases, 24 were HPV-18 positive cases and 20 were HPV negative. CSCC and non-tumor biopsies from CSCC patients as well as C-33A cell lines were used. Cells were transfected with MAGI2-AS3 and CDK6 expression vectors as well as with MAGI2-AS3 siRNA to analyze gene interactions. Cell cycle analysis was performed to analyze the effects of transfections on cell cycle progression. qPCR and western blot were applied to analyze gene expression. Paired t-test and ANOVA (one-way) combined with Tukey test were used for data comparisons. Survival analysis was performed by plotting and comparing survival curves.

**Results:**

qRT-PCR results showed that CDK6 and MAGI2-AS3 were both up-regulated in CSCC and positively correlated with each other. MAGI2-AS3 and CDK6 expression was not significantly affected by HPV infections. High levels of MAGI2-AS3 were associated with the poor survival of CSCC patients. In CSCC cells, MAGI2-AS3 over-expression up-regulated CDK6, while MAGI2-AS3 siRNA down-regulated CDK6. In CCK-8 assay, MAGI2-AS3 and CDK6 over-expression led to increased proliferation rate of CSCC cells by reducing CDK6 levels, while MAGI2-AS3 siRNA didn’t. In addition, CDK6 over-expression attenuated the effect of MAGI2-AS3 siRNA silencing.

**Conclusions:**

In conclusion, MAGI2-AS3 promoted CSCC cell proliferation by up-regulating CDK6.

## Background

Cervical cancer is the 2nd most common type of gynecological tumors worldwide, and cervical squamous cell carcinoma (CSCC) accounts for 80–90% of cervical cancer [1, 2]. Although the mortality and the morbidity rates have dropped significantly during the past several decades, early diagnosis is still technically challenging and about 50% of patients are diagnosed at advanced stages [3, 4]. Once tumor metastasis occurred, only less than 40% of CSCC patients can live longer than 5 years [5]. Infections of human papillomavirus (HPV) are major causes of CSCC. With the popularization of HPV vaccination, incidence and mortality of CSCC has dropped significantly in past century [1–4]. However, they are not sufficient for cervical cancer development. There are also HPV negative CSCC cases with more aggressive nature [6].

Altered cell cycle progression plays pivotal roles in the development and progression of CSCC [7]. Comparing to normal cells, cell phase transition in cancer cells is accelerated and inhibition of cell cycle progression is considered as a promising approach for cancer treatment [8]. Cyclin-dependent kinases, or CDKs, such as CDK6, bind cyclins to mostly participate in G1 phase regulation [9]. In vitro cell experiments showed that inhibition of CDK6 by its inhibitor LEE011 induced G1 phase arrest, thereby suppressing cancer cell proliferation in leukemia [10]. It is known that CDK6 activity can be regulated by long non-coding RNAs (> 200 nt, lncRNAs) [11], which are a group of RNA transcripts with no protein-coding capacity but able to regulate downstream gene expression to participate in cellular processes, such as transcription and DNA repair [12]. In a recent study, it has been reported that lncRNA MAGI2-AS3 regulated breast cancer cell proliferation through the modulation of Fas and Fas ligand [13]. This study aims to investigate the role of MAGI2-AS3 in CSCC.

## Methods

### Study patients and follow-up

Sixty-four CSCC patients (36 to 68 years, 46.3 ± 5.1 years) out of 136 CSCC patients, admitted to Shengjing Hospital affiliated to China Medical University from June 2010 to October 2013, were included in the present study. Inclusion criteria were: 1) no therapies received before admission; 2) new CSCC cases; 3) willing to complete follow-up; 4) completed 5-year follow-up. Exclusion criteria: 1) recurrent CSCC; 2) medical records showed histories of malignancies; 3) therapies were initiated; 4) patients died of other causes; 5) patients lost during follow-up. Based on their medical records, there were 20 HPV-16 positive cases, 24 HPV-18 positive cases, and 20 HPV negative cases. Based on AJCC (American Joint Committee on Cancer) staging criteria, the 64 CSCC patients included 10, 14, 24, and 16 cases at AJCC stage I-IV, respectively. All patients signed informed consent. Shengjing Hospital Ethics Committee approved this study.

### CSCC tissues and cells

All patients donated CSCC and adjacent (2 cm around tumors) non-tumor tissues, which were collected by performing a biopsy. All tissue specimens were tested by histopathological exams. C-33A human CSCC cell line (ATCC, USA) was included in this study. Cells were cultivated at 37 °C with 5% CO_2_ in Eagle’s Minimum Essential Medium (10% FBS).

### Lipofectamine 2000-mediated cell transfections

MAGI2-AS3 and CDK6 expression vectors were constructed using the pcDNA3.1 vector (RIBOBIO, Guangzhou, China). MAGI2-AS3 siRNA and siRNA negative control (NC) was bought from GenePharma (Shanghai, China). C-33A cells were harvested at the confluence of 70–80% and 10 nM MAGI2-AS3 or CDK6 expression vector (empty pcDNA3.1 vector as NC group) or 35 nM MAGI2-AS3 siRNA (siRNA NC as NC group) were transfected into 10^5^ cells in a 2 ml cell suspension. This experiment included un-transfected cells as control (C). The interval between transfection and the subsequent experiment was 24 h.

### Total RNA and qPCR

All tissue specimens were grounded in liquid nitrogen to prepare fine tissue powder. An amount of 0.01 g tissue powder or 10^5^ cells were mixed with 0.4 ml Trizol reagent (Invitrogen, USA) to extract total RNAs. AMV Reverse Transcriptase XL (Takara, USA) was used to perform reverse transcriptions with 3000 ng total RNA in a 20 μl reaction mixture. The synthesized cDNA was diluted 10 times, and SYBR Green qPCR kit (Bio-Rad, USA) was used to prepare qPCR reaction mixtures with 1 μl cDNA in a 20 μl volume. The expression of MAGI2-AS3 and CDK6 mRNAs was normalized using GAPDH as endogenous control. All PCR reactions were repeated 3 times and data were analyzed using the 2^-ΔΔCT^ method.

### CCK-8 assay

C-33A cells were collected at 24 h after transfections, and 1 ml Eagle’s Minimum Essential Medium (10% FBS) was mixed with 3 × 10^4^ cells (after trypsinization) to make cell suspensions. In a 96-well plate, cells were cultivated (0.1 ml cell suspension in each well) in a 37 °C and 5% CO_2_ incubator. CCK-8 solution (10 μl, Sigma-Aldrich, USA) was added into each well at 3 h before the termination of cell culture. OD values were measured at 450 nm.

### Cell cycle analysis

After trypsinization, 10^5^ C-33A cells were washed with pre-cold PBS. Cells were participated and pre-cold ethanol (75%) was used to dissolve the cells, followed by incubation at 4 °C for 4 h. After that, cells were washed with cold PBS again. After washing, cells were stained with BD Pharmingen™ PI/RNase for 30 min at 25 °C, followed by flow cytometer at different cell cycle phases (G1, S, and G2). In each sample, 10^5^ events were counted.

### Western blot

After trypsinization, C-33A cells were counted and 10^5^ cells were resuspended in 0.4 ml RIPA solution (RIBOBIO) to extract total proteins. All protein samples were quantified by performing a BCA assay. After protein denaturation in boiling water for 10 min, electrophoresis was performed using 10% -PAGE gel, followed by protein transfer to PVDF membranes. All membranes were incubated with 5% non-fat milk for 25 at 24 °C. Rabbit polyclonal primary antibodies of GAPDH (ab37168, 1:1100, Abcam) and CDK1 (ab151247, 1:1100, Abcam) were used to incubate the membranes for 1 h at 4 °C. HRP (IgG) goat anti-rabbit secondary antibody (ab6721, 1:900, Abcam) was used to further incubate with the membranes for 2 h at 24 °C. ECL (Sigma-Aldrich, USA) and ImageJ v1.46 software were used for signal development and normalization, respectively.

### Data analysis

All data presented in this paper were mean values calculated using data from 3 replicates. The paired t-test was used to compare CSCC and non-tumor tissues. One-way ANOVA was used to compare multiple patient and cell groups. Correlations were analyzed using linear regression. Based on survival data, survival curves were plotted using Kaplan-Meier plotter and compared by the log-rank test. A *p*-value < 0.05 was considered statistically significant.

## Results

### CDK6 and MAGI2-AS3 were up-regulated and correlated in CSCC

**Fig. 1:**
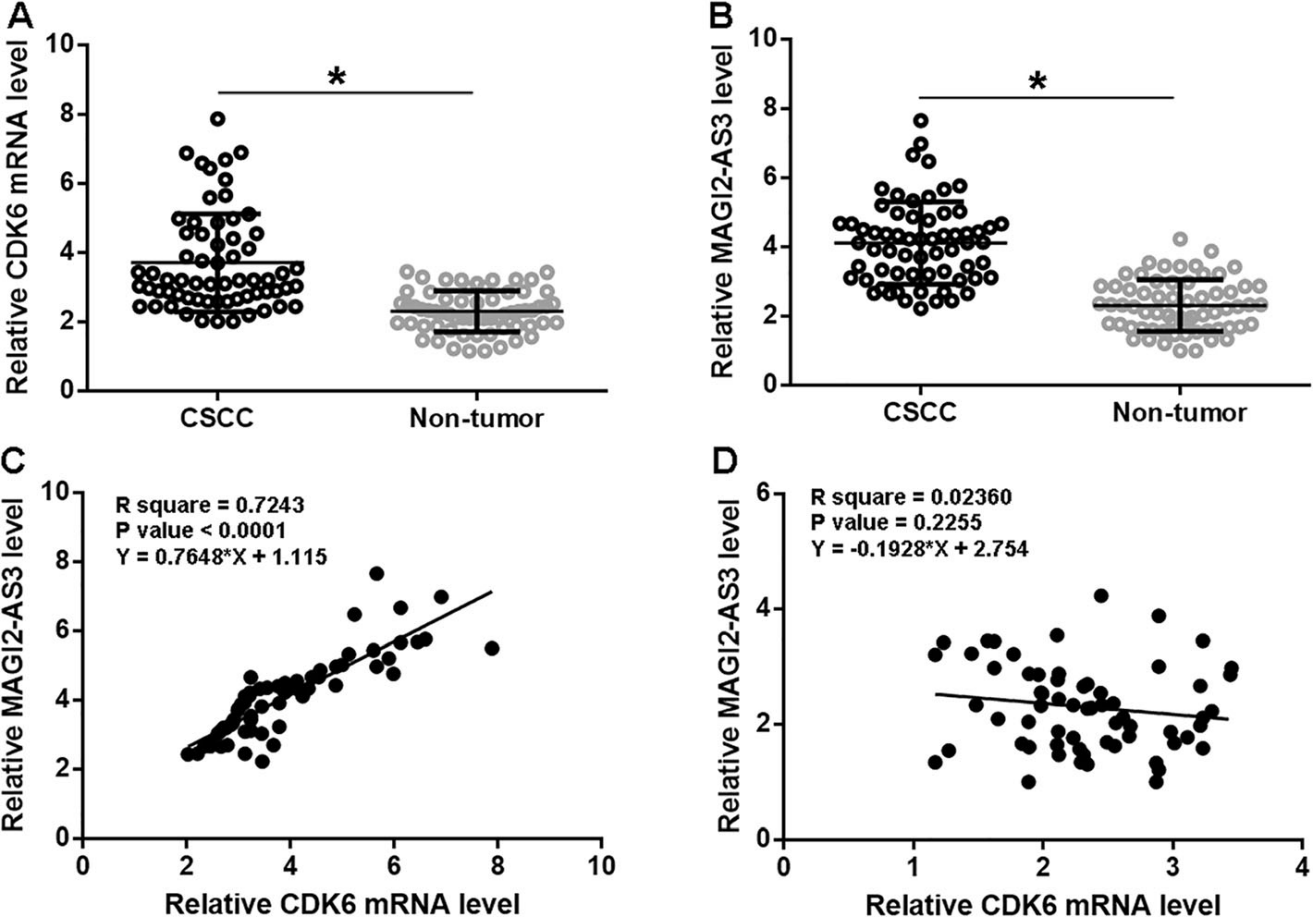
CDK6 and MAGI2-AS3 were up-regulated and correlated in CSCC. CDK6 (**a**) and MAGI2-AS3 (**b**) expression levels were measured by qPCR and compared (CSCC vs. non-tumor) by paired t test. This experiment was performed 3 times. The correlation between CDK6 and MAGI2-AS3 CSCC tissues (**c**) and non-tumor tissues (**d**) was analyzed by linear regression. All values were mean values

CDK6 and MAGI2-AS3 in CSCC and non-tumor tissues collected from 64 CSCC patients were first detected by RT-qPCR. Expression levels of those two were compared by a paired t-test. It was observed that levels of CDK6 (Fig. 1a) and MAGI2-AS3 (Fig. 1b) expressions were significantly higher in CSCC tissues comparing to non-tumor tissues (*p* < 0.05). It was observed that CDK6 expression was positively correlated with MAGI2-AS3 expression in CSCC tissues (Fig. 1c), but not in non-tumor tissues (Fig. 1d).

### MAGI2-AS3 and CDK6 expression was not significantly affected by HPV infections

**Fig. 2:**
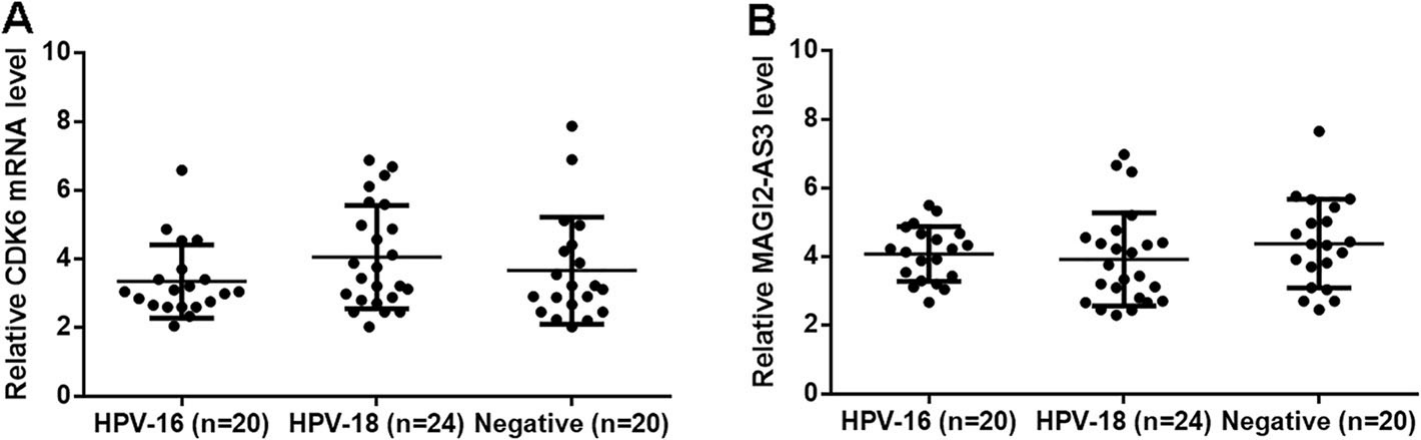
MAGI2-AS3 and CDK6 expression was not significantly affects by HPV infections. The 64 CSCC patients included 20 HPV-16 positive cases, 24 HPV-18 positive cases and 20 HPV negative cases. Expression levels of CDK6 (**a**) and MAGI2-AS3 (**b**) in CSCC tissues measured by qPCR (above mentioned) were compared among 3 groups of patients by performing ANOVA (one-way) and Tukey test. All values were mean values

The 64 CSCC patients included 20 HPV-16 positive cases, 24 HPV-18 positive cases, and 20 HPV negative cases. Expression levels of CDK6 and MAGI2-AS3 in CSCC tissues were measured by qPCR and compared by one-way ANOVA and Tukey test. It was observed that expression levels of CDK6 (Fig. 2a) and MAGI2-AS3 (Fig. 2b) were not different among the 3 groups.

### High levels of MAGI2-AS3 were associated with the poor survival of CSCC patients

**Fig. 3:**
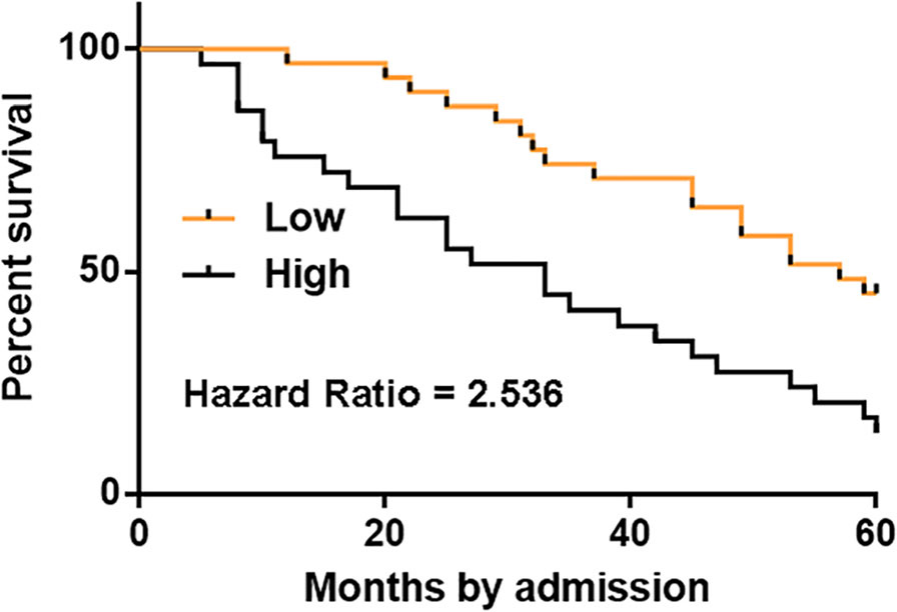
High levels of MAGI2-AS3 were associated with the poor survival of CSCC patients. Survival curves of both high (*n* = 31) and low (*n* = 33) MAGI2-AS3 level groups were plotted using K-M plotter and compared by log-rank test

The 64 CSCC patients were grouped into high (*n* = 31) and low (*n* = 33) MAGI2-AS3 level groups with the cutoff value identified by Youden’s index. Using K-M plotter, survival curves of both high (*n* = 31) and low (*n* = 33) MAGI2-AS3 level groups were plotted. The log-rank test was used to compare survival curves. It was observed that the overall survival rate of high MAGI2-AS3 level group during the 5-year follow-up was significantly higher than that of low MAGI2-AS3 level group (*p* = 0.024, Fig. 3).

### MAGI2-AS3 positively regulated CDK6 in C-33A cells

**Fig. 4:**
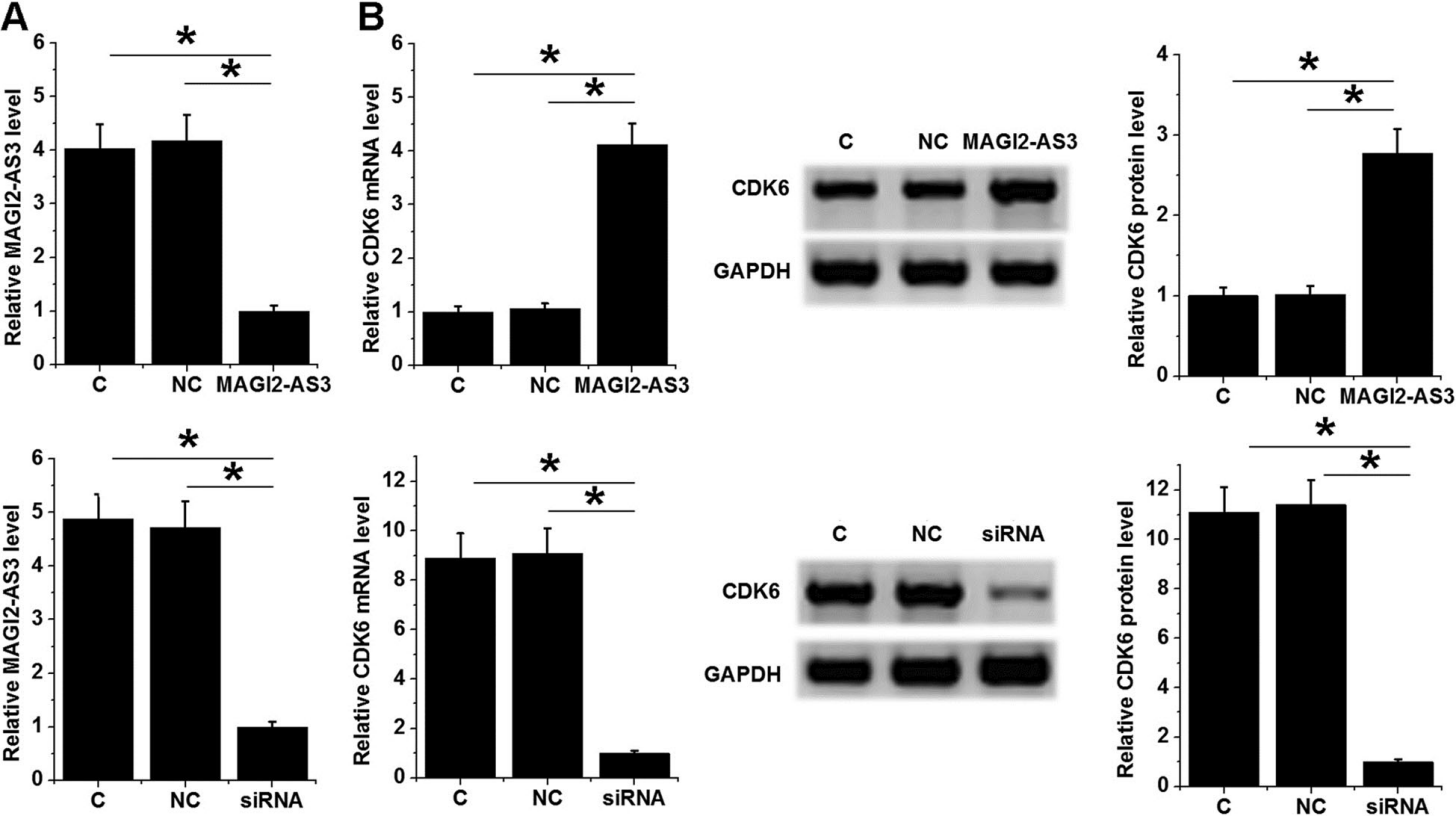
MAGI2-AS3 positively regulated CDK6 in C-33A cells. C-33A cells were transfected with MAGI2-AS3 expression vector and siRNA, and the over-expression and knockdown of MAGI2-AS3 comparing to C and NC groups were confirmed by RT-qPCR at 24 h post-transfection (**a**). The effects of MAGI2-AS3 over-expression and siRNA silencing on CD6 at mRNA and protein levels were analyzed by qPCR and western-blot, respectively (**b**). Experiments were performed 3 times. All values were mean values

C-33A cells were transfected with MAGI2-AS3 expression vector and siRNA, and the over-expression and knockdown of MAGI2-AS3 were confirmed by RT-qPCR at 24 h post-transfection (Fig. 4a, *p* < 0.05). Moreover, MAGI2-AS3 over-expression up-regulated CDK6 at mRNA and protein levels, while MAGI2-AS3 siRNA silencing down-regulated them (Fig. 4b, *p* < 0.05).

### MAGI2-AS3 regulated cell proliferation by affecting cell cycle through CDK6

**Fig. 5:**
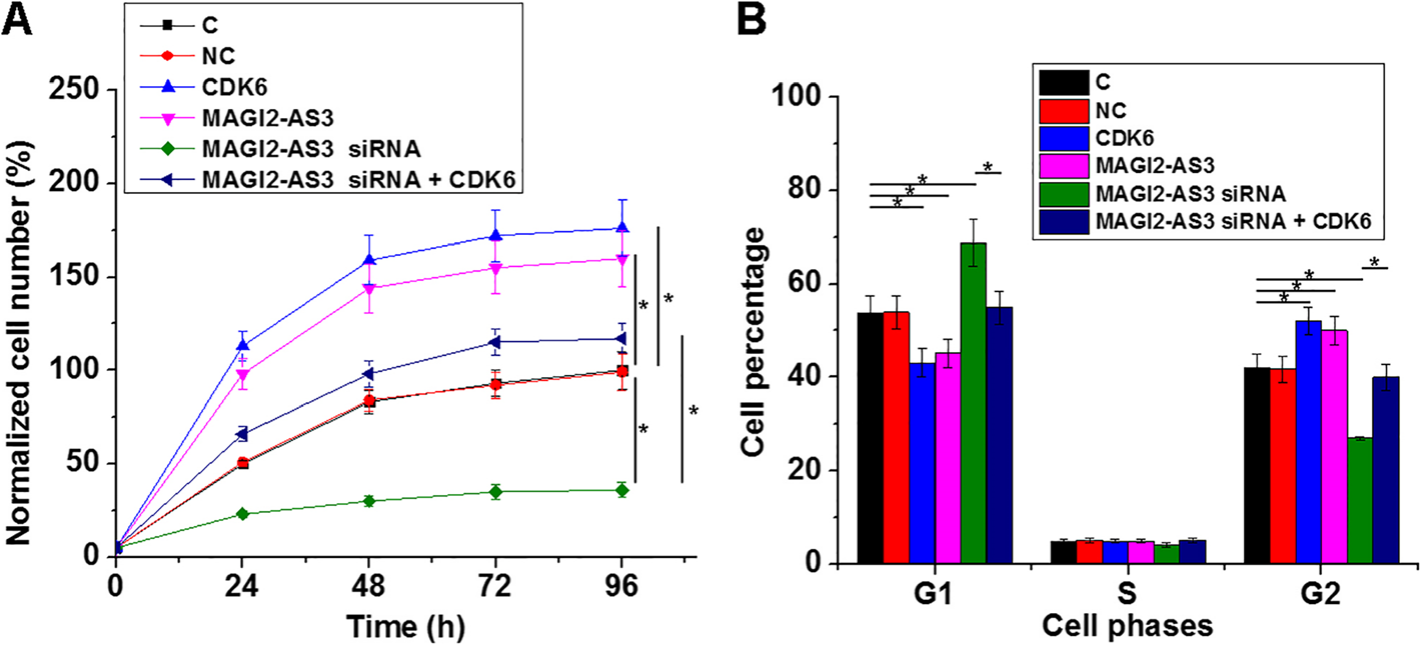
MAGI2-AS3 regulated cell proliferation by affecting cell cycle through CDK6. The effects of MAGI2-AS3 and CDK6 over-expression as well as MAGI2-AS3 siRNA silencing on cell proliferation (**a**) and cell cycle progression (**b**) were analyzed by performing CCK-8 assay and cell cycle assay, respectively. All experiments were repeated 3 times. All values were mean values

The CCK-8 assay showed that MAGI2-AS3 and CDK6 overexpression in CSCC led to increased proliferation of CSCC cells, compared to C and NC groups, while MAGI2-AS3 siRNA didn’t (Fig. 5a, *p* < 0.05). The cell cycle analysis showed that MAGI2-AS3 and CDK6 overexpression in CSCC, compared to C and NC groups, led to decreased percentage of cells at G1, and opposite effects on G2 phase cell percentage were also observed (Fig. 5b, *p* < 0.05). Moreover, CDK6 over-expression attenuated the effects of MAGI2-AS3 siRNA silencing on cell proliferation and cell cycle progression (*p* < 0.05).

## Discussion

The role of MAGI2-AS3 in CSCC has not been reported. This study mainly investigated the role of MAGI2-AS3 in CSCC. We found that MAGI2-AS3 was up-regulated in CSCC and regulates cell cycle progression. The actions of MAGI2-AS3 in this regulation are at least partially mediated by CDK6.

It has been reported that lncRNA MAGI2-AS3 regulated breast cancer cell proliferation through the modulation of Fas and Fas ligand [13]. Besides that, Wang et al. also reported that MAGI2-AS3 suppressed bladder cancer progression by sponging miR-15b-5p to regulate the expression of CCDC19 [14]. Those two studies showed the tumor-suppressive roles of MAGI2-AS3 in two different types of cancer. Interestingly, our study showed that MAGI2-AS3 promoted the proliferation and cell cycle progression in CSCC, indicating its oncogenic roles. The controversial observation is possibly due to the different pathogenesis of different types of cancer. However, patients and the cell line used in this study may also affect the results.

Our study showed that high expression levels of MAGI2-AS3 were closely correlated with the poor survival of CSCC patients. At present, effective therapeutic approaches for most advanced cancer including CSCC are not available and patients’ survival is poor [15]. Even after active chemotherapy, such as Bevacizumab, more than 50% of the patients will die of this disease within 5 years after admission. Therefore, accurate prognostic markers may be used to improve the survival of CSCC patients through several different ways, such as to guide the development of individualized care program and to help the selection of certain therapies [16]. Our data showed that detection of MAGI2-AS3 expression may assist the prognosis of CSCC. However, future studies are needed to further test the accuracy.

We showed that MAGI2-AS3 positively regulated CDK6. The possible mechanism remains unknown. Our preliminary data showed that MAGI2-AS3 may serve as the sponge of miR-320 and miR-186 (data not shown), which can directly target CDK6 [17, 18]. We will perform in-depth investigations on the possible interaction among those factors and report our findings in future studies.

It is worth noting that the sample size is small and the single-cell line used in this study may provide biased results. Our future studies will include more patients and cell lines to further confirm our conclusions.

In conclusion, MAGI2-AS3 was up-regulated in CSCC and promoted CSCC cell proliferation and cell cycle progression by positively regulating CDK6.

## Data Availability

The datasets used and/or analyzed during the current study are available from the corresponding author on reasonable request.

## Abbreviations

CSCC: Cervical squamous cell carcinoma
HPV: Human papillomavirus
CDKs: Cyclin-dependent kinases
